# Supplementary material for: The Influence of Lateral Size and Oxidation of Graphene Oxide on Its Chemical Reduction and Electrical Conductivity of Reduced Graphene Oxide
Source: Molecules. 2022 Nov 14;27(22):7840. doi: 10.3390/molecules27227840 (PMC9696217; doi:10.3390/molecules27227840)
Supplement: Supplementary file 1 [file molecules-27-07840-s001.zip › molecules-1988970-supplementary.pdf]

Supporting Information

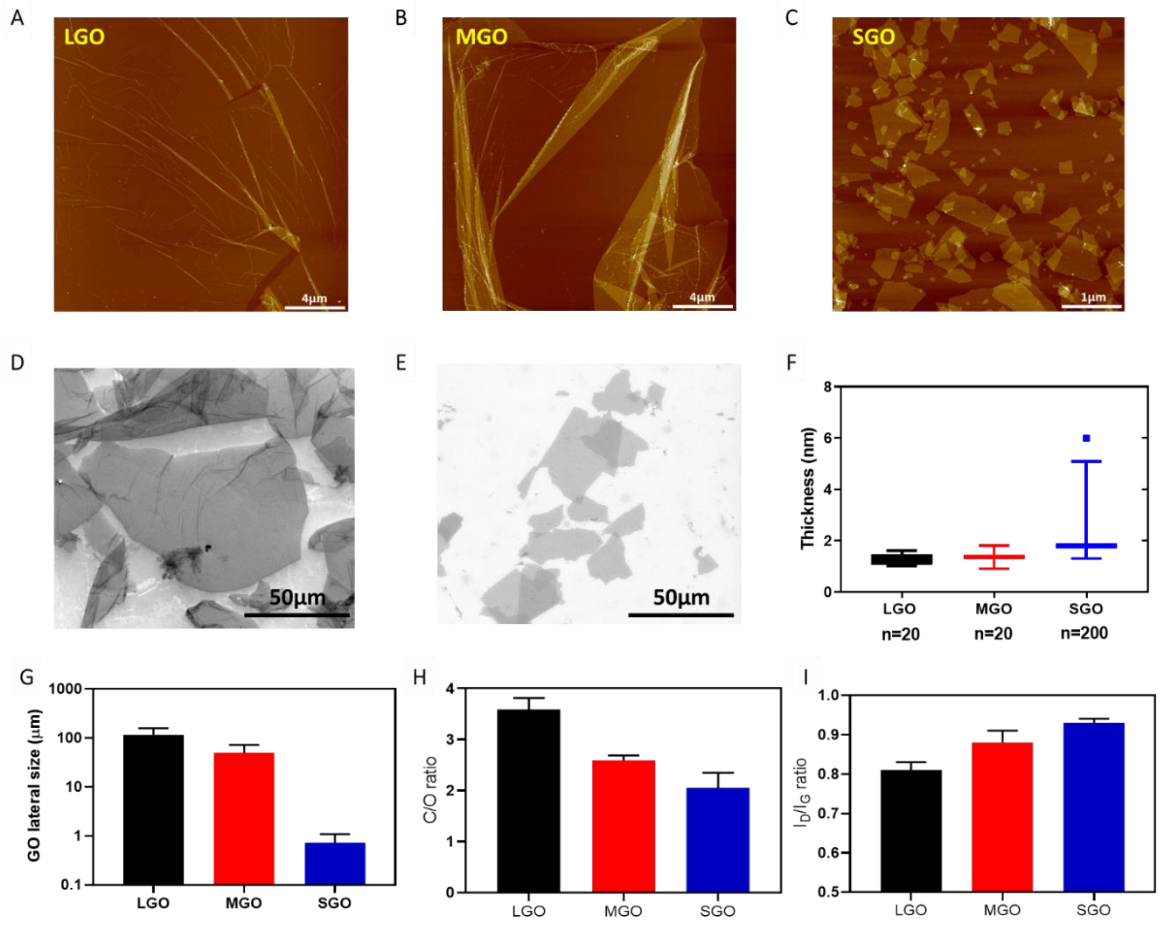

Figure S1: **Morphological and chemical properties of LGO (black), MGO (red) and SGO (blue) used in this study.** Two-dimensional AFM images of (a) LGO, (b) MGO, and (c) SGO. FESEM images of (d) LGO, and (e) MGO. SGO was too small to be analysed using FESEM imaging. (f) Box-plot of GO thickness based on AFM analysis, and the population of monolayer GO in SGO, MGO and LGO samples were 91%, 98% and 99% respectively. Error bars represent standard error mean (SEM), n represents total number of GO sheets measured. (g) Lateral size of GOs based on FESEM imaging. (h) C/O ratio of all GOs determined using XPS. (i) I_D/I_G ratio of GOs obtained from Raman spectroscopy. For the detailed characterisations please see the previous study [1].

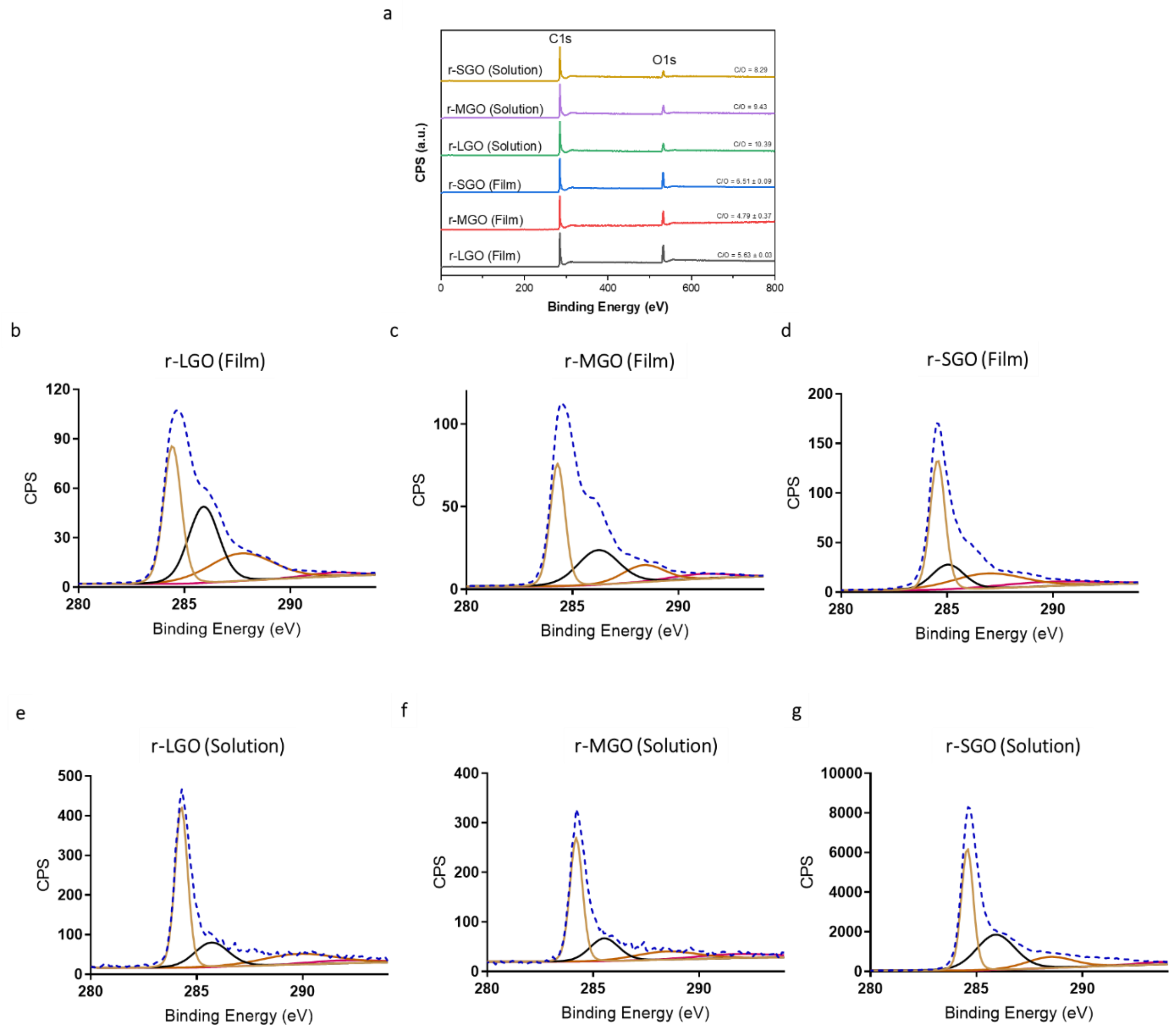

Figure S2. Various chemical and physical characterization of GO and rGO samples reduced by ascorbic acid. (a) XPS general survey. XPS C1s deconvolution spectra of (b) r-LGO (Film). (c) r-MGO (Film). (d) r-SGO (Film). (e) r-LGO (Solution). (f) r-MGO (Solution). (g) r-SGO (Solution).

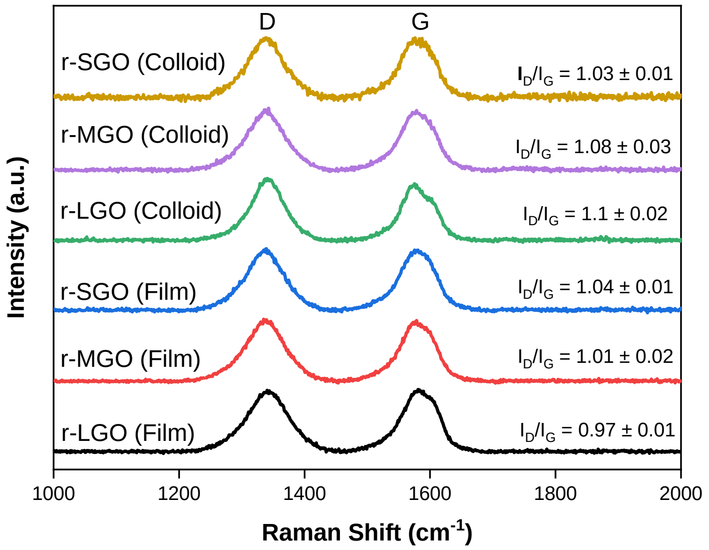

Figure S3. Raman spectroscopy of various rGO films reduced using L-ascorbic acid as reductant. Error bars represent standard error of mean (SEM), n = 3.

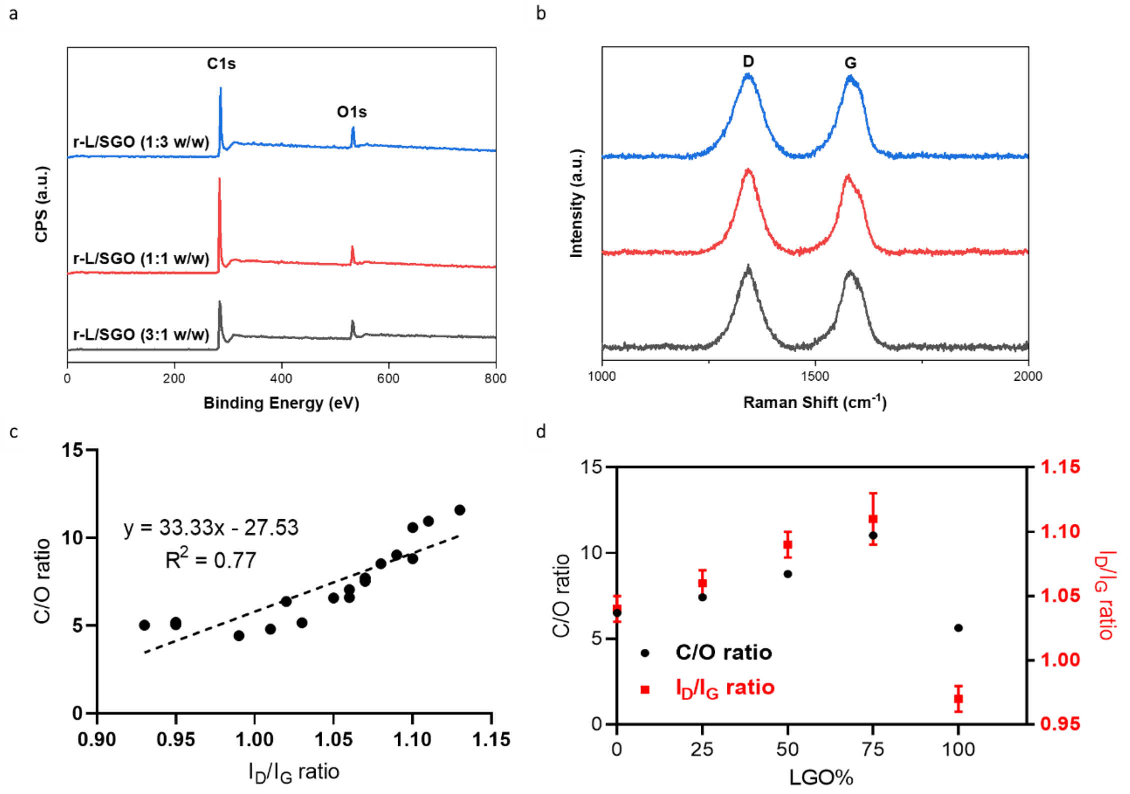

Figure S4. Physicochemical characterisation of rGO films. The films were made from using LGO and SGO at different ratios, and then reduced using L-ascorbic acid as reductant. (a) XPS general survey. (b) Raman spectra. (c) Correlation between C/O ratio and I_D/I_G value of rGO films with the solid line represents a linear regression fit. (d) LGO content dependence of C/O ratio and I_D/I_G ratio of rGO films. All error bars represent standard error of mean, n = 3.

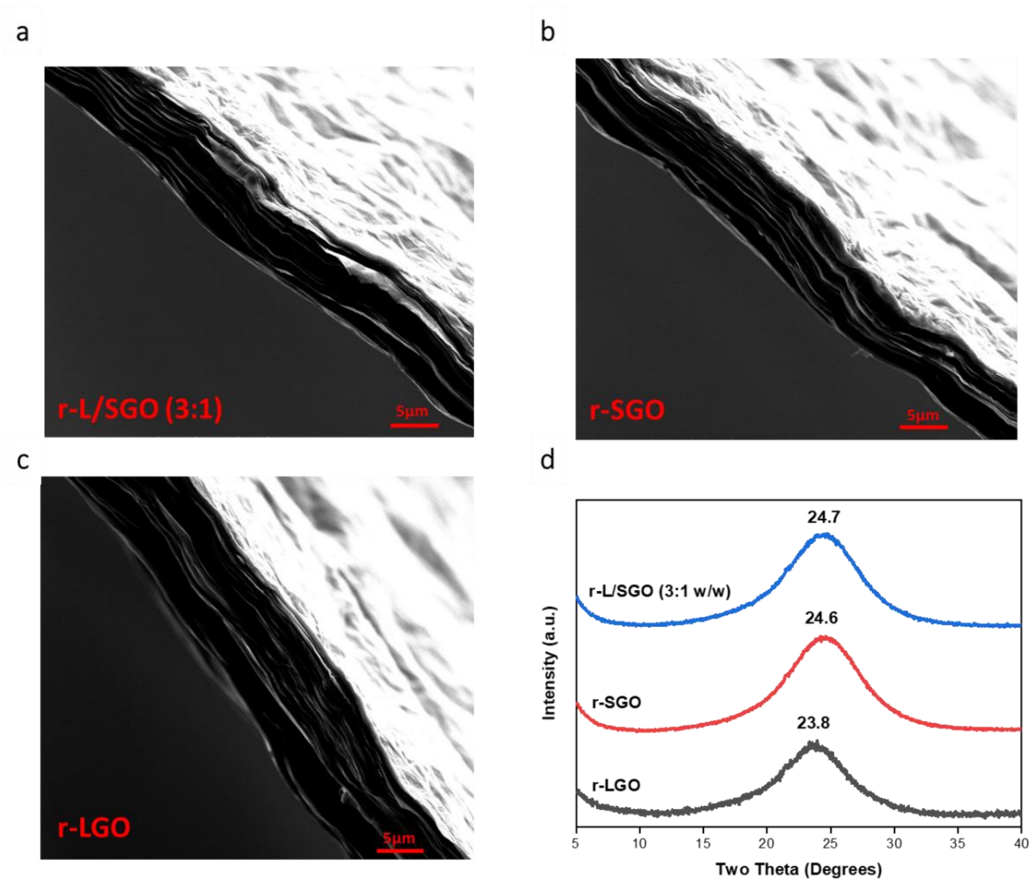

Figure S5: Structural characterization of rGO films prepared using L-ascorbic acid as reductant. FESEM film images of (a) r-L/SGO (3:1 w/w), (b) r-SGO, and (c) r-LGO film. (d) XRD spectra of various rGO films.

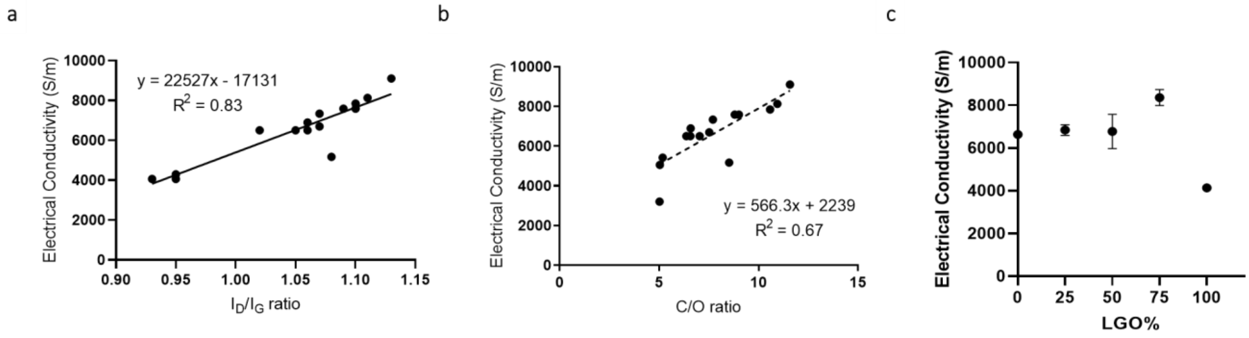

Figure S6: The electrical conductivity of rGO films prepared using L-ascorbic acid as reductant. Correlation between the electrical conductivity with (a) I_D/I_G ratio, (b) C/O ratio, and (c) percentage of LGO in the mixture of L/SGO. The electrical conductivity was derived from contactless resistance values measured at 3 different points of each film. The solid line in (a) and (b) represents a linear regression analysis.

Table S1: Summary of XPS C1s deconvolution results of different GO samples.

	C=C/C-C (%)	C-O (%)	O-C=O (%)	π-π (%)
LGO	47.16	31.79	16.42	4.63
MGO	42.71	36.4	18.72	2.17
SGO	33.79	42.89	21.4	1.92

Table S2: Summarised chemical characterisation of varying rGO samples, synthesized by L-ascorbic acid, from XPS general survey and deconvolution of C1s spectra. Error bars represent standard error of mean (SEM), n = 3.

	C/O ratio	C=C/C-C (%)	C-O (%)	O-C=O (%)	π-π (%)
r-LGO (Solution)	10.87 ± 0.84	50.73	21.72	20.26	7.29
r-MGO (Solution)	9.02 ± 0.21	46.8	24.18	22.66	6.36
r-SGO (Solution)	7.75 ± 0.28	39.72	29.97	26.64	3.68
r-LGO (Film)	6.51 ± 0.09	50.08	24.33	21.15	4.44
r-MGO (Film)	4.79 ± 0.37	44.86	26.52	23.63	4.99
r-SGO (Film)	5.63 ± 0.03	49.28	27.75	21.3	1.67

Table S3: Chemical composition of various r-L/SGO and r-L/MGO films, formed by a mixture of LGO or MGO with SGO films (w/w), reduced by HI acid reductant. XPS was used to obtain these results. Error bars represent standard error of mean with n = 3.

	C/O ratio	C=C/C-C (%)	C-O (%)	O-C=O (%)	π-π (%)
r-L/SGO (3:1 w/w)	20.37 ± 0.19	63.49	10.86	12.69	12.96
r-L/SGO (1:1 w/w)	18.99 ± 0.38	60.18	14.68	12.52	12.62
r-L/SGO (1:3 w/w)	16.8 ± 0.49	56.14	21.11	13.06	9.69
r-M/SGO (3:1 w/w)	18.18 ± 0.44	58.2	20.01	14.73	7.06
r-M/SGO (1:1 w/w)	16.17 ± 0.26	54.26	22.65	14.61	8.47
r-M/SGO (1:3 w/w)	15.11 ± 0.31	53.27	24.38	14.52	7.83

Table S4: Summary of Raman and X-ray diffraction (XRD) characterisation of rGO films chemically reduced by HI acid and L-ascorbic acid reductants.

	Film	I_D/I_G ratio	002 Diffraction Peak (2θ)	D-spacing (nm)
HI reductant	r-L/SGO (3:1)	1.34 ± 0.01	25.1	0.354
	r-L/SGO (1:1)	1.32 ± 0.02	24.7	0.36
	r-L/SGO (1:3)	1.29 ± 0.02	24.35	0.365
	r-SGO	1.27 ± 0.01	24.4	0.364
	r-LGO	1.14 ± 0.01	24.0	0.37
AA reductant	r-L/SGO (3:1)	1.11 ± 0.01	24.7	0.36
	r-L/SGO (1:1)	1.09 ± 0.02	24.7	0.36
	r-L/SGO (1:3)	1.05 ± 0.03	24.6	0.361
	r-SGO	1.04 ± 0.01	24.6	0.361
	r-LGO	0.97 ± 0.01	23.8	0.373

Table S5. Summary and comparison of rGO properties of this study with that reported in literature.

Sample Type	Reduction Conditions	C/O ratio	I_D/I_G ratio	D-spacing (nm)	Electrical Conductivity ($S\ m^{-1}$)	Reference
RGO	Hydrazine, 150 °C, 12h	11	-	0.370 (24°)	16000	[2]
RGO	Hydrazine, 95 °C, 1h	-	-	0.341 (26.1°)	35100	[3]
RGO	L-ascorbic acid, 95 °C, 15min	12.5	-	-	7700	[4]
RGO	Ammonia borane, 66 °C, 12h	9.8	1.7	-	20300	[5]
RGO	Hydrazine, 100 °C, 3h	11.7	1.6	0.370 (23.9°)	-	[6]
RGO	200 °C, 1h	-	-	0.369 (24.1°)	41000	[7]
RGO	HI, 25 °C, 24h	7.68		0.364 (24.47°)	40900	[8]
RGO	FeI_2, 95 °C, 6h	-	1.5	0.369 (24.1°)	55088	[9]
RGO	Hydrazine, 1100 °C, 3h	12.5	1.6	-	55000	[10]
RGO	Hydrazine, Ammonia, 95 °C, 1h	-	-	-	7200	[11]
RGO	HI, 95 °C, 1h	-	-	-	36400	[12]
RGO	HI, 100 °C, 1h	12	-	0.357 (24.4°)	29800	[13]
RGO	1100 °C in Argon/H_2 gas	-	1.2	0.347 (25.4°)	55000	[14]
RGO	Ethylene, 900 °C in Argon/H_2 gas, 1h	-	1.4	-	35000	[15]
RGO	HI-AcOH, 40 °C, 40h	6.7	1.1	0.362 (24.6°)	30400	[16]
Graphite	None	-	-	0.330 (26.7°)	84500	[16]
RGO	HI, 80 °C, 1h	20.37	1.34	0.354 (25.1°)	85283	This work

References

1. Sim, H. J.; Xiao, P.; Lu, H., Pyrenebutyric acid-assisted room-temperature synthesis of large-size monolayer graphene oxide with high mechanical strength. *Carbon* **2021,** 185, 224-233.
2. Park, S.; An, J.; Jung, I.; Piner, R. D.; An, S. J.; Li, X.; Velamakanni, A.; Ruoff, R. S., Colloidal Suspensions of Highly Reduced Graphene Oxide in a Wide Variety of Organic Solvents. *Nano Letters* **2009,** 9, (4), 1593-1597.
3. Chen, H.; Müller, M. B.; Gilmore, K. J.; Wallace, G. G.; Li, D., Mechanically Strong, Electrically Conductive, and Biocompatible Graphene Paper. *Advanced Materials* **2008,** 20, (18), 3557-3561.
4. Fernández-Merino, M. J.; Guardia, L.; Paredes, J. I.; Villar-Rodil, S.; Solís-Fernández, P.; Martínez-Alonso, A.; Tascón, J. M. D., Vitamin C Is an Ideal Substitute for Hydrazine in the Reduction of Graphene Oxide Suspensions. *The Journal of Physical Chemistry C* **2010,** 114, (14), 6426-6432.
5. Pham, V. H.; Hur, S. H.; Kim, E. J.; Kim, B. S.; Chung, J. S., Highly efficient reduction of graphene oxide using ammonia borane. *Chemical Communications* **2013,** 49, (59), 6665-6667.
6. Sevilla, M.; Ferrero, G. A.; Fuertes, A. B., Aqueous Dispersions of Graphene from Electrochemically Exfoliated Graphite. *Chemistry – A European Journal* **2016,** 22, (48), 17351-17358.
7. Yu, P.; Tian, Z.; Lowe, S. E.; Song, J.; Ma, Z.; Wang, X.; Han, Z. J.; Bao, Q.; Simon, G. P.; Li, D.; Zhong, Y. L., Mechanically-Assisted Electrochemical Production of Graphene Oxide. *Chemistry of Materials* **2016,** 28, (22), 8429-8438.

8. Chen, J.; Li, Y.; Huang, L.; Jia, N.; Li, C.; Shi, G., Size Fractionation of Graphene Oxide Sheets via Filtration through Track-Etched Membranes. *Adv Mater* **2015,** 27, (24), 3654-60.
9. Liu, C.; Hao, F.; Zhao, X.; Zhao, Q.; Luo, S.; Lin, H., Low temperature reduction of free-standing graphene oxide papers with metal iodides for ultrahigh bulk conductivity. *Scientific Reports* **2014,** 4, (1), 3965.
10. Mattevi, C.; Eda, G.; Agnoli, S.; Miller, S.; Mkhoyan, K. A.; Celik, O.; Mastrogiovanni, D.; Granozzi, G.; Garfunkel, E.; Chhowalla, M., Evolution of Electrical, Chemical, and Structural Properties of Transparent and Conducting Chemically Derived Graphene Thin Films. *Advanced Functional Materials* **2009,** 19, (16), 2577-2583.
11. Li, D.; Müller, M. B.; Gilje, S.; Kaner, R. B.; Wallace, G. G., Processable aqueous dispersions of graphene nanosheets. *Nature Nanotechnology* **2008,** 3, (2), 101-105.
12. Liu, Y.; Wu, X.; Tian, Y.; Zhou, X.; Yu, B.; Zhang, Q.; Du, R.; Fu, Q.; Chen, F., Largely enhanced oxidation of graphite flakes via ammonium persulfate-assisted gas expansion for the preparation of graphene oxide sheets. *Carbon* **2019,** 146, 618-626.
13. Pei, S.; Zhao, J.; Du, J.; Ren, W.; Cheng, H.-M., Direct reduction of graphene oxide films into highly conductive and flexible graphene films by hydrohalic acids. *Carbon* **2010,** 48, (15), 4466-4474.
14. Wang, X.; Zhi, L.; Müllen, K., Transparent, Conductive Graphene Electrodes for Dye-Sensitized Solar Cells. *Nano Letters* **2008,** 8, (1), 323-327.
15. López, V.; Sundaram, R. S.; Gómez-Navarro, C.; Olea, D.; Burghard, M.; Gómez-Herrero, J.; Zamora, F.; Kern, K., Chemical Vapor Deposition Repair of Graphene Oxide: A Route to Highly-Conductive Graphene Monolayers. *Advanced Materials* **2009,** 21, (46), 4683-4686.
16. Moon, I. K.; Lee, J.; Ruoff, R. S.; Lee, H., Reduced graphene oxide by chemical graphitization. *Nature Communications* **2010,** 1, (1), 73.
